# The serrulatane diterpenoid natural products RAD288 and RAD289 stimulate properties of olfactory ensheathing cells useful for neural repair therapies

**DOI:** 10.1038/s41598-018-28551-2

**Published:** 2018-07-06

**Authors:** Mo Chen, Marie-Laure Vial, Johana Tello Velasquez, Jenny A. K. Ekberg, Rohan A. Davis, James A. St John

**Affiliations:** 1 Griffith Institute for Drug Discovery, Griffith University, Brisbane, 4111 QLD Australia; 2 Menzies Health Institute Queensland, Griffith University, Southport, 4222 QLD Australia; 3 Clem Jones Centre for Neurobiology and Stem Cell Research, Griffith University, Brisbane, 4111 QLD Australia

## Abstract

Olfactory ensheathing cells (OECs) are being trialled for cell transplantation therapies for neural repair as they have unique properties which can enhance neuron regeneration. However, improvements in cell viability, proliferation and migration are needed to enhance therapeutic outcomes. Growth factors can enhance cell activity, but they can also induce side effects as they can act on numerous cell types. An alternative approach is to identify natural products (NPs) that more selectively activate specific cell functions. We have examined two pure NPs, 3-acetoxy-7,8-dihydroxyserrulat-14-en-19-oic acid (RAD288) and 3,7,8-trihydroxyserrulat-14-en-19-oic acid (RAD289) isolated from the Australian plant *Eremophila microtheca*. We determined that RAD288 and RAD289 stimulated the viability and proliferation of OECs in two-dimensional cultures and increased cell viability in three-dimensional spheroids. Both compounds also enhanced OEC-mediated phagocytosis of neural debris. However, only RAD288 stimulated migration of OECs, demonstrating that key structural changes to the compound can dramatically affect the resultant cellular action. In addition, cell-type specific action is highlighted by the result that neither compound stimulated the viability of Schwann cells which are a closely-related glial cell type. Therefore, these small molecules may have high potential for selective activation of specific therapeutically-useful activities of OECs for transplantation therapies to repair the nervous system.

## Introduction

Olfactory ensheathing cells (OECs), a subtype of glia located within the olfactory mucosa and the olfactory bulb, facilitate the regeneration of olfactory neurons throughout the life of mammals^1^. Due to their ability to promote nerve regeneration, the transplantation of OECs is a promising approach for neural repair therapies including spinal cord injury (SCI) repair. When transplanted into the injured spinal cord, OECs have been reported to promote remyelination of damaged neurons^2^, enhance axonal regeneration^3^ and interact with astrocytes which facilitate the migration of OECs into the lesion site^4^. However, the survival of transplanted OECs is limited^5,6^ and the subsequent migration of OECs into the host tissue is slow^7^.

In order to improve therapeutic outcomes after transplantation of OECs, a prioritised strategy is to improve proliferation and migration rates and to improve the functional activity of OECs. Within the olfactory system after an injury, OECs migrate ahead of axons and enhancing the migration of OECs can improve axon growth after major injury^8^. OECs are also the major phagocytic cell of the olfactory system and remove cell debris after injury^9^, which is also of high benefit for neural repair therapies. Therefore, improving the function and distribution of OECs is crucial in a cell-based neural repair therapy. OECs express a range of growth factors including nerve growth factor, brain-derived neurotrophic factor and glia cell-line derived neurotrophic factor (GDNF)^10^ and the addition of exogenous growth factors can stimulate glial cell growth. Indeed, OECs that have been genetically engineered to enhance secretion of GDNF are reported to have improved functional outcomes in a spinal cord injury model^11^. However, genetically engineering transplanted cells with elevated levels of growth factors may potentially produce unknown long term consequences^12^. An alternative approach for neural repair is to administer exogenous growth factors into the injury site to enhance proliferation of transplanted glial cells^13^. However, some growth factor treatments have been reported to be associated with serious side effects, including constant back pain and hyperinnervation of cerebral blood vessels^14–16^ as the growth factors can also stimulate endogenous cells within the host. In an effort to identify further avenues to stimulate transplanted cells to improve neural regeneration, screening of natural products (NPs) provides a wealth of opportunity to discover compounds that mimic the effects of neurogenic factors, particularly those that stimulate activity at low concentrations and with fewer side-effects.

**Figure 1:**
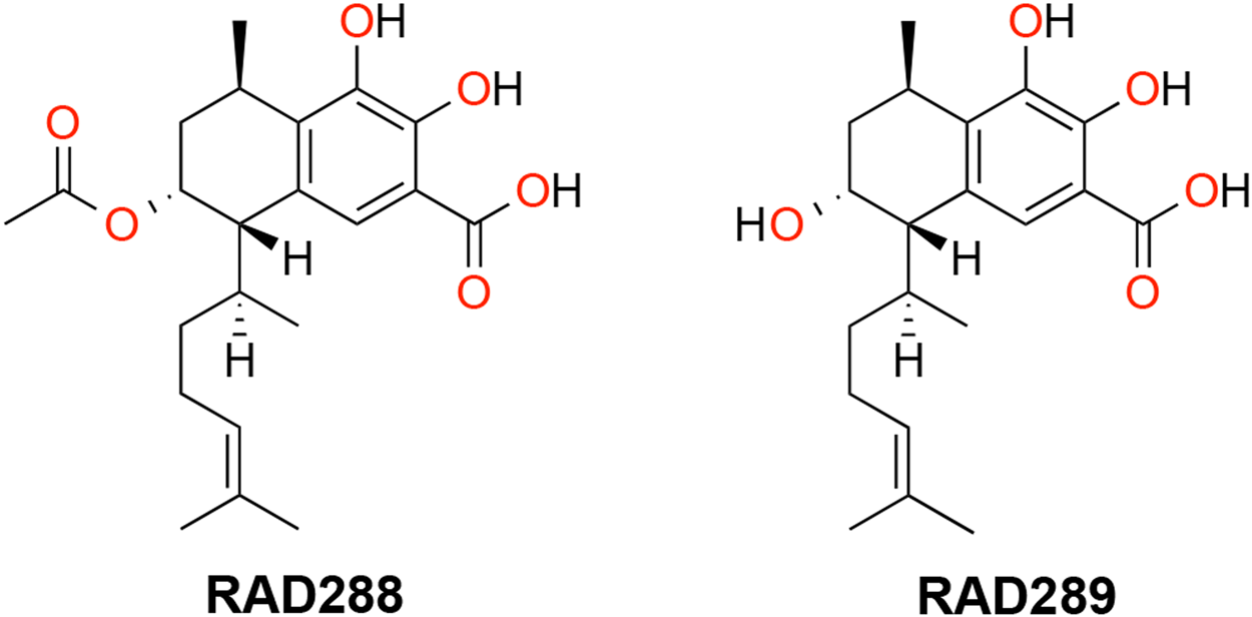
Structure of RAD288 (3-acetoxy-7,8-dihydroxyserrulat-14-en-19-oic acid) and RAD289 (3,7,8-trihydroxyserrulat-14-en-19-oic acid).

In this study, we examined the effect of the NPs 3-acetoxy-7,8-dihydroxyserrulat-14-en-19-oic acid (RAD288) and 3,7,8-trihydroxyserrulat-14-en-19-oic acid (RAD289) (Fig. 1) that have been isolated from the aerial parts of the Australian desert plant *Eremophila microtheca*^17^. *Eremophila* species have commonly been used for medicinal purposes by Australian Aboriginal people^18^, for instance, to treat colds and influenza^19^. RAD288 and RAD289 have been previously shown to display anti-bacterial properties^17^, which is also of relevance for neural repair therapies, since these compounds may aid in the reduction of bacterial infections. We therefore tested whether RAD288 and RAD289 would also stimulate the proliferation, migration, and phagocytic activity of mouse OECs (mOECs). In this study, we found that the structurally similar compounds RAD288 and RAD289 stimulated the activity of OECs leading to a significant increase in proliferation, morphological changes and phagocytic activity, but that only RAD288 stimulated migration. When tested on the closely related glial cell type, Schwann cells, the compounds had no effect on proliferation. These results indicate that RAD288 and RAD289 stimulate specific but different activities of OECs, and are active on select cell types. These serrulatane diterpenoids are therefore potentially useful for improving glial cell activity in cell transplantation therapies.

## Results

### RAD288 and RAD289 increase cell proliferation of mOECs

**Figure 2:**
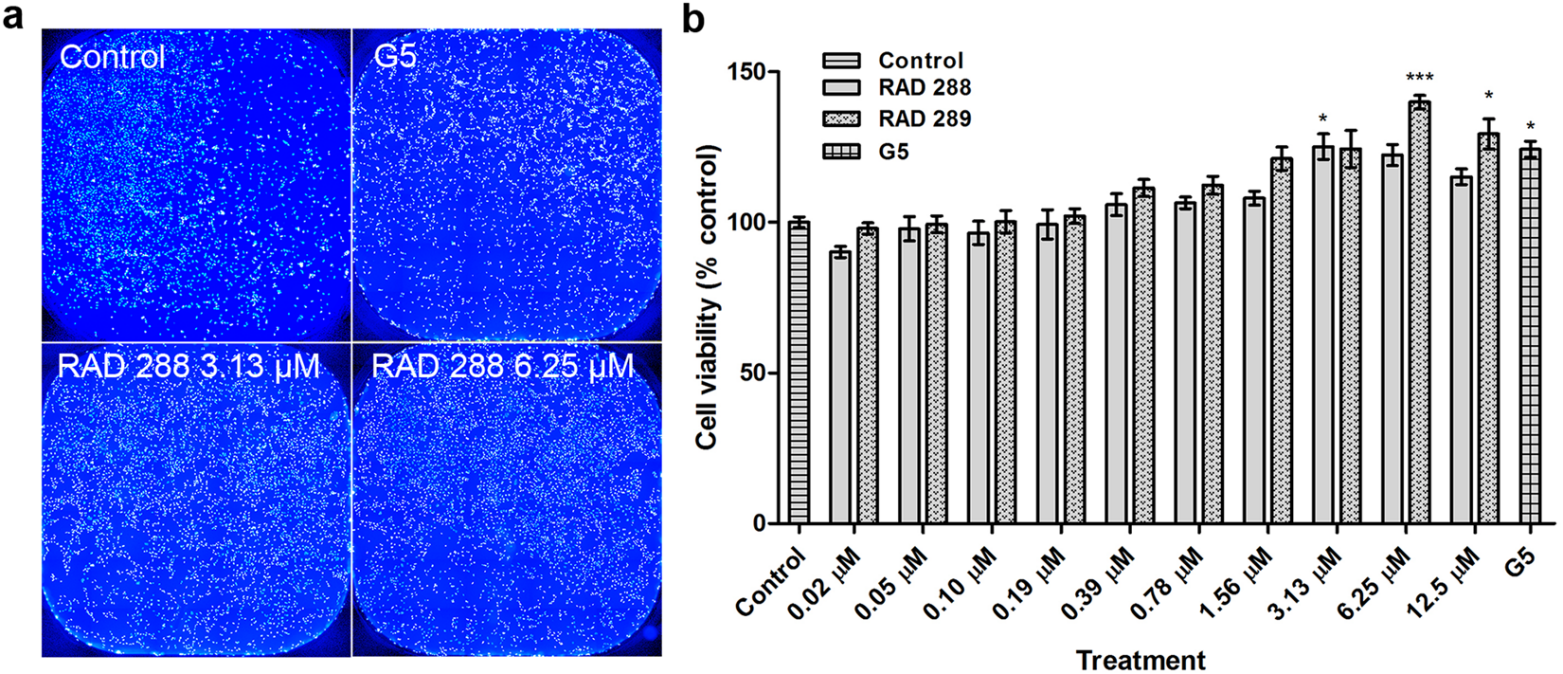
Effects on metabolic activity and proliferation of mOECs after treatments with RAD288 and RAD289. (**a**) Representative images of mOECs after drug exposure. Nuclei were stained with Hoechst. Scale bar = 100 µm. (**b**) Cell viability of mOECs exposed to 0.2% dimethyl sulfoxide (control), 1% G5 Supplement, RAD288 (0.02–12.5 µM) and RAD289 (0.02–12.5 µM) using the resazurin metabolic activity indicator. Triplicate wells were used in three separate experiments, mean ± SEM. ***p < 0.001, *p < 0.05, Student’s t-test.

To determine whether RAD288 and RAD289 affect the cell viability and proliferation of mOECs, cells were treated with a range of concentrations from 0.02 to 12.5 µM of RAD288 and RAD289 for 24 h. Cell viability was assessed using the resazurin reduction assay. For a positive control, we used the commercial product G5 Supplement (ThermoFisher Scientific) which contains a mixture of factors including biotin (100 mg/L), basic FGF (0.5 mg/L), EGF (1.0 mg/L), human transferrin (5000 mg/L), insulin (500 mg/L), hydrocortisone (0.36 mg/L) and selenite (0.52 mg/L). Identifying a single natural compound that can stimulate OEC growth and activity to a similar or better level than G5 Supplement would indicate the natural compound is potentially useful for production of OECs *in vitro* or for stimulating OECs *in vivo* after transplantation. The positive control G5 Supplement alone exhibited a significant 24.31% increase in mOEC cell viability (p < 0.05) compared to the control treatment. We also found that both RAD288 and RAD289 promoted mOEC cell viability (Fig. 2a). For RAD288, the peak increase in viability was at a concentration of 3.13 µM with a 25.13% increase (p < 0.05); the other concentrations tested did not show any significant effects compared to the negative control DMSO (p > 0.05) (Fig. 2b). For RAD289, the peak increase was at a concentration of 6.25 µM with a 39.94% increase in the viability (p < 0.001) (Fig. 2b); RAD289 at 12.5 µM also significantly increased viability (p < 0.05) (Fig. 2b). As the resazurin assay is a measure of viability and an indirect measurement of proliferation, we performed a cell count of each well using the Operetta High-Content Imaging System and the Harmony software. RAD288 at 3.13 µM increased cell numbers by 22.89% (p < 0.05) while RAD289 at 6.25 µM increased cell numbers by 32.87% (p < 0.05), which confirms the resazurin reduction assay results. Therefore, RAD288 (3.13 µM) and RAD289 (6.25 µM) enhance both viability and proliferation of mOECs.

**Figure 3:**
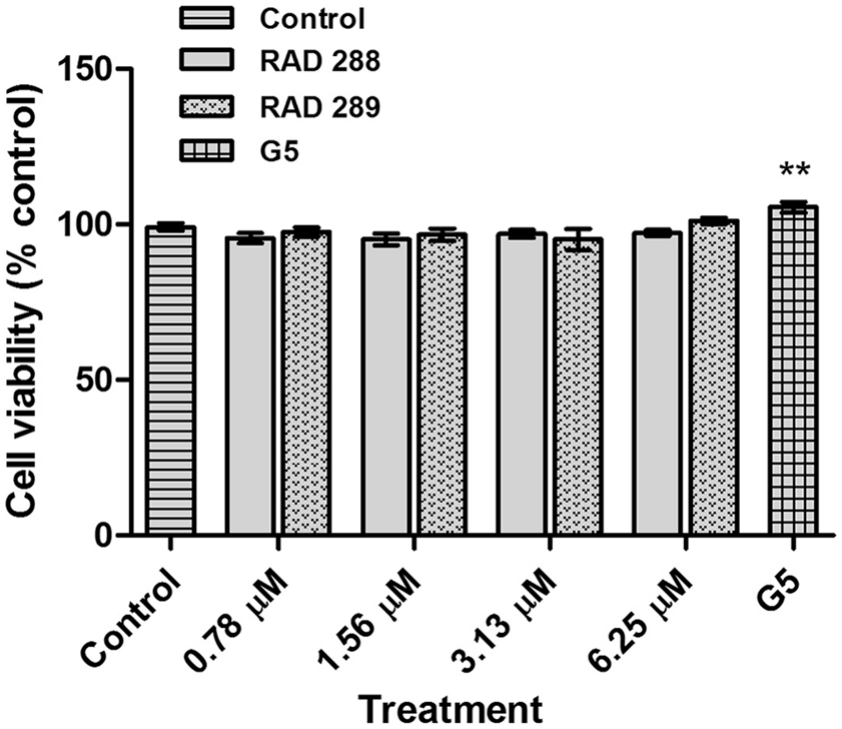
RAD288 and RAD289 do not affect Schwann cell viability. Cell viability of Schwann cells exposed to 0.2% dimethyl sulfoxide (control), 1% G5 supplement, RAD288 (0.78–6.25 µM) and RAD289 (0.78–6.25 µM) using the resazurin metabolic activity indicator. Triplicate wells were used in three separate experiments, mean ± SEM, **p < 0.01, Student’s t-test.

Schwann cells, which are glial cells in the general peripheral nervous system (PNS), play an important role in tissue repair process after PNS injury^20^ and are also a target for use in neural repair therapies. To determine if Schwann cells also responded to RAD288 and RAD289, Schwann cells were treated with a range of concentrations from 0.78 to 6.25 µM of RAD288 and RAD289 for 24 h. We observed that both natural products did not affect the viability of Schwann cells (p > 0.05) in comparison with the negative control. In contrast, G5 supplement treatment increased cell viability by 10% (p < 0.01) (Fig. 3). Consequently, further investigations on RAD288 and RAD289 were only performed on mOECs.

### RAD288 and RAD289 induce morphological alterations in mOECs

**Figure 4:**
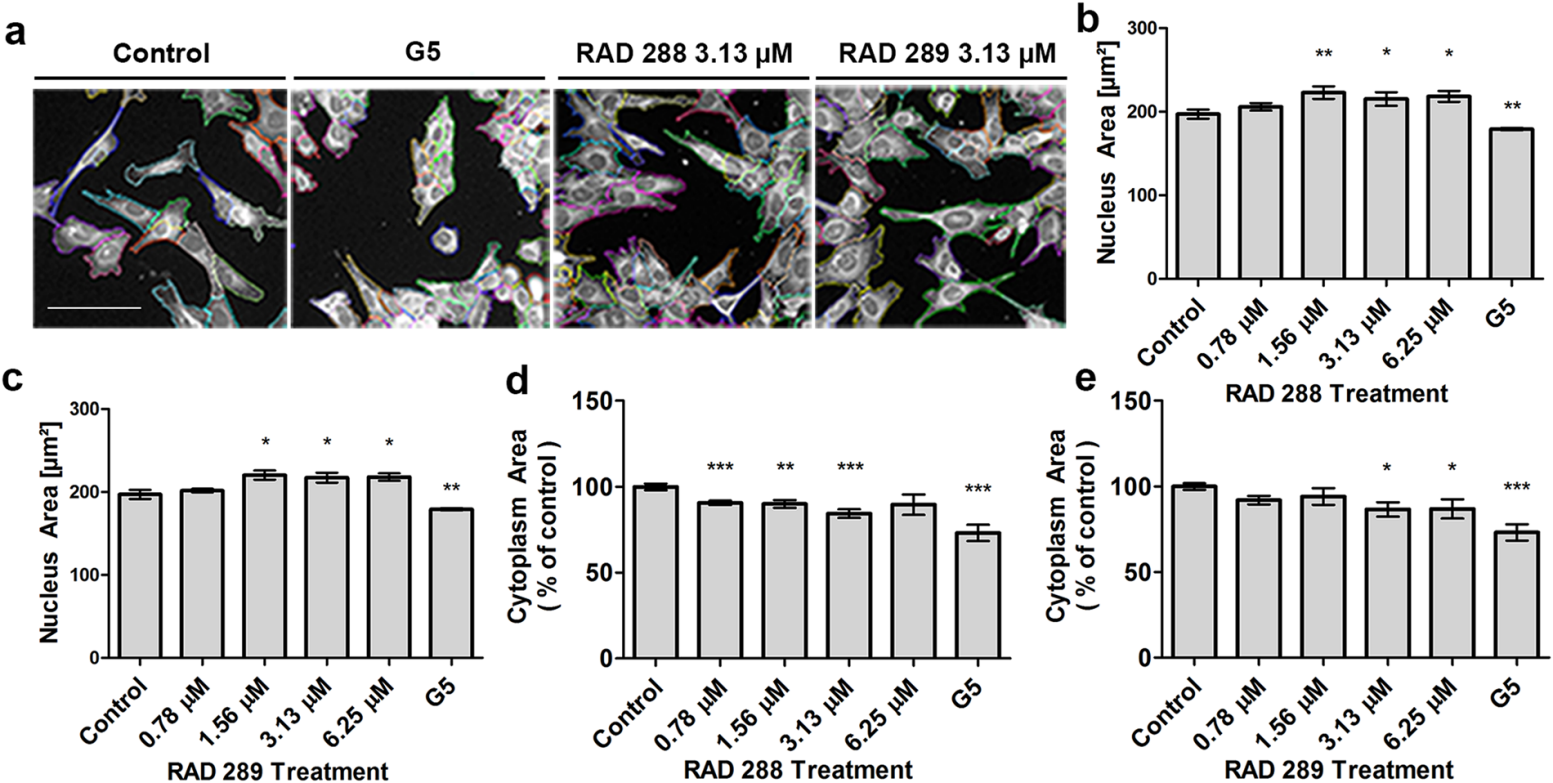
Effect of RAD288 and RAD289 on morphology of mOECs. (**a**) Representative images of mOECs after drug exposure. Images were segmented with Harmony software using Find Cytoplasm method. Scale bar = 100 µm. (**b**) Nucleus area of mOECs after treatment with several concentrations of RAD288. (**c**) Nucleus area of mOECs after treatment with a range of concentrations of RAD289. (**d**) Cytoplasm area of mOECs after treatment with several concentrations of RAD288. (**e**) Cytoplasm area of mOECs after treatment with a range of concentrations of RAD289. Negative control (0.2% DMSO) and positive control (1% G5 Supplement) were used. Triplicate wells were used in three separate experiments. Results represent the mean +/− SEM. ***p < 0.001, **p < 0.01, *p < 0.05, Student’s t-test.

Interestingly, RAD288 and RAD289 produced dramatic changes in the cell shape of mOECs. These effects were clearly visible after 24 h treatment with a range of concentrations from 0.78 to 6.25 µM of RAD288 and RAD289 in comparison with the negative control DMSO (Fig. 4a). Indeed, the nucleus size slightly increased after treatment with these compounds at concentrations of 1.56, 3.13 and 6.25 µM (p < 0.05) (Fig. 4b,c). In addition, a decrease in the cytoplasm area was observed after treatments with RAD288 with a 10% decrease at 0.78 µM (p < 0.001), a 10% decrease at 1.56 µM (p < 0.01) and a 15% decrease at 3.13 µM (p < 0.001) (Fig. 4d). For RAD289 at 3.13 and 6.25 µM, a 13% decrease after exposure also occurred (p < 0.05) (Fig. 4e). Treatments with G5 Supplement alone showed a 10% and 25% decrease in nucleus area and cytoplasm area, respectively (Fig. 4).

**Figure 5:**
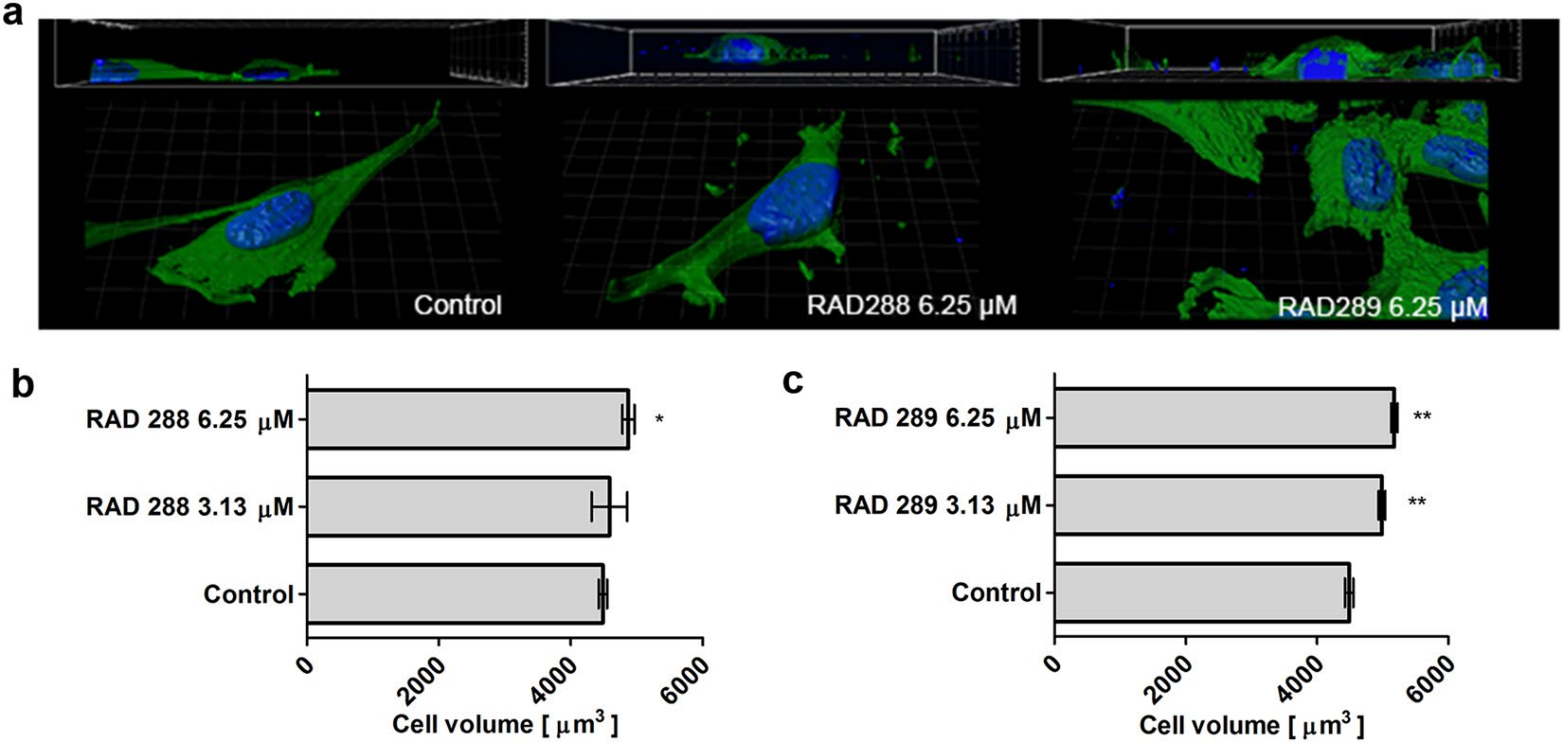
Analysis of mOEC cytoplasmic volume after treatment with RAD288 and RAD289. (**a**) Representative images of mOECs after drug exposure generated by confocal microscopy and Imaris software. (**b**) Cytoplasmic volume of mOECs after treatment with RAD288 at 3.13 and 6.25 µM. (**c**) Cytoplasmic volume of mOECs after treatment with RAD289 at 3.13 and 6.25 µM. Triplicate wells were used in three separate experiments, mean ± SEM. **p < 0.01, *p < 0.05, Student’s t-test.

To further analyse the previous results, 3D morphological studies were carried out on mOECs after treatment with RAD288 and RAD289 at concentrations of 3.13 and 6.25 µM. In contrast to the flat morphology observed when treated with the negative control (DMSO only), mOECs exhibited a well-rounded shape after treatments with RAD288 and RAD289 (Fig. 5a). A 2.2% increase in cytoplasmic volume on mOECs treated with 6.25 µM of RAD288 was observed (p < 0.05) (Fig. 5b). Our results also showed that mOEC cytoplasmic volume increased by 11% (p < 0.05) and 15% (p < 0.01) after treatments with 3.13 and 6.25 µM of RAD289, respectively (Fig. 5c).

### RAD288 promotes mOEC migration

**Figure 6:**
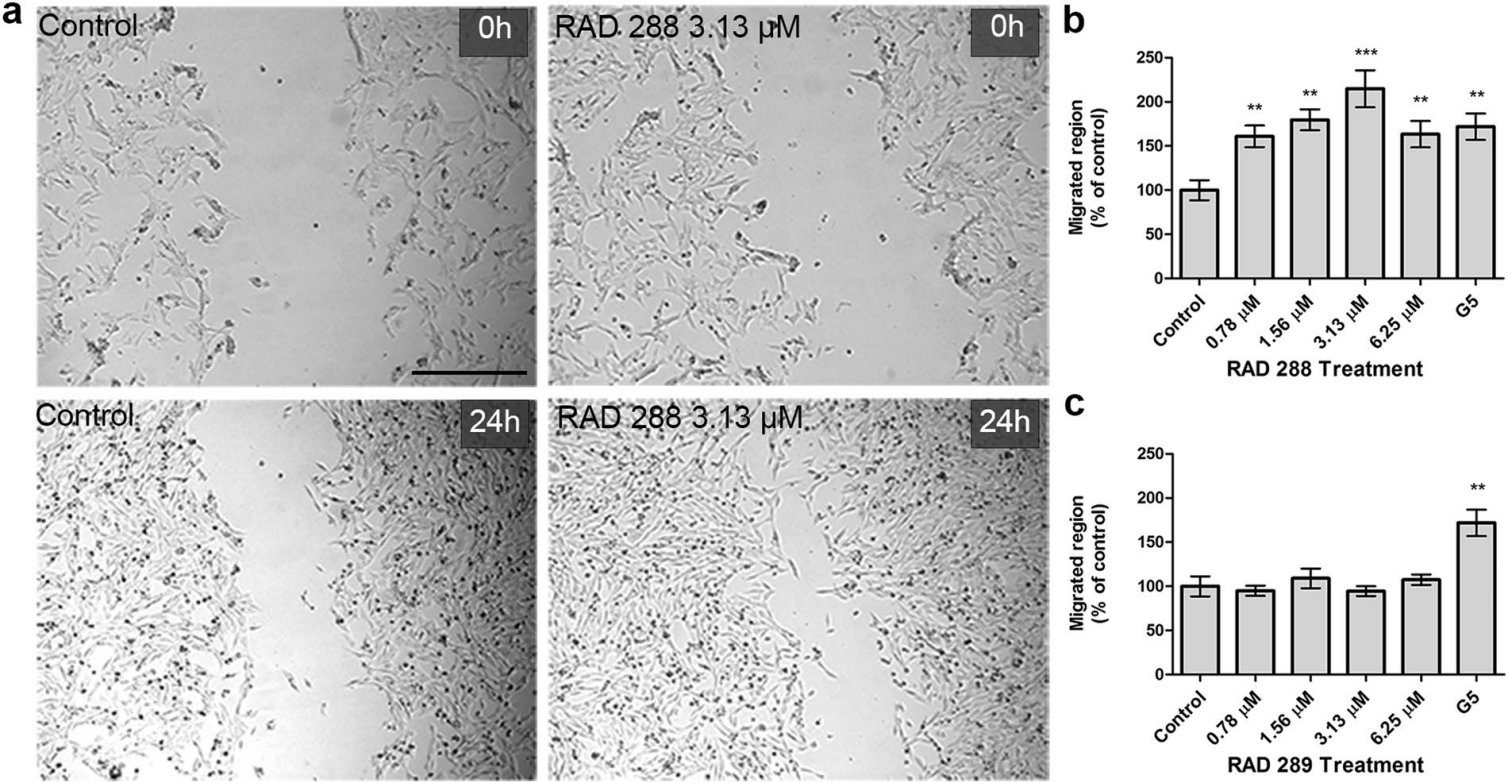
RAD288 enhances cell migration of mOECs *in vitro*. (**a**) Cell migration was detected using wound scratch assay. Representative wound closure images after drug exposure. Scale bar = 500 µm. (**b**) Migrated region after treatments with different concentrations of RAD288 expressed as the percentage of control. (**c**) Migrated region after treatments with different concentrations of RAD289 expressed as the percentage of control. Values are presented as means ± SEM of five independent experiments. ***p < 0.001, **p < 0.01, Mann-Whitney U test.

To examine the effect of RAD288 and RAD289 on mOEC migration, an *in vitro* scratch assay was performed using time-lapse microscopy. We observed that mOECs migrated further into the scratch after 24 h treatment with RAD288 in comparison with the negative control DMSO (Fig. 6a). Indeed, the migrated region was increased by 61% (p < 0.01), 80% (p < 0.01), 115% (p < 0.001) and 63% (p < 0.01) after treatment with RAD288 at concentrations of 0.78, 1.56, 3.13 and 6.25 µM, respectively (Fig. 6b). In contrast, RAD289 did not show any effect on the migration ability of mOECs (Fig. 6c). The migrated region increased by 72% (p < 0.01) after treatment with G5 Supplement alone (Fig. 6b,c).

### RAD288 and RAD289 stimulate phagocytosis of neuron debris by mOECs

**Figure 7:**
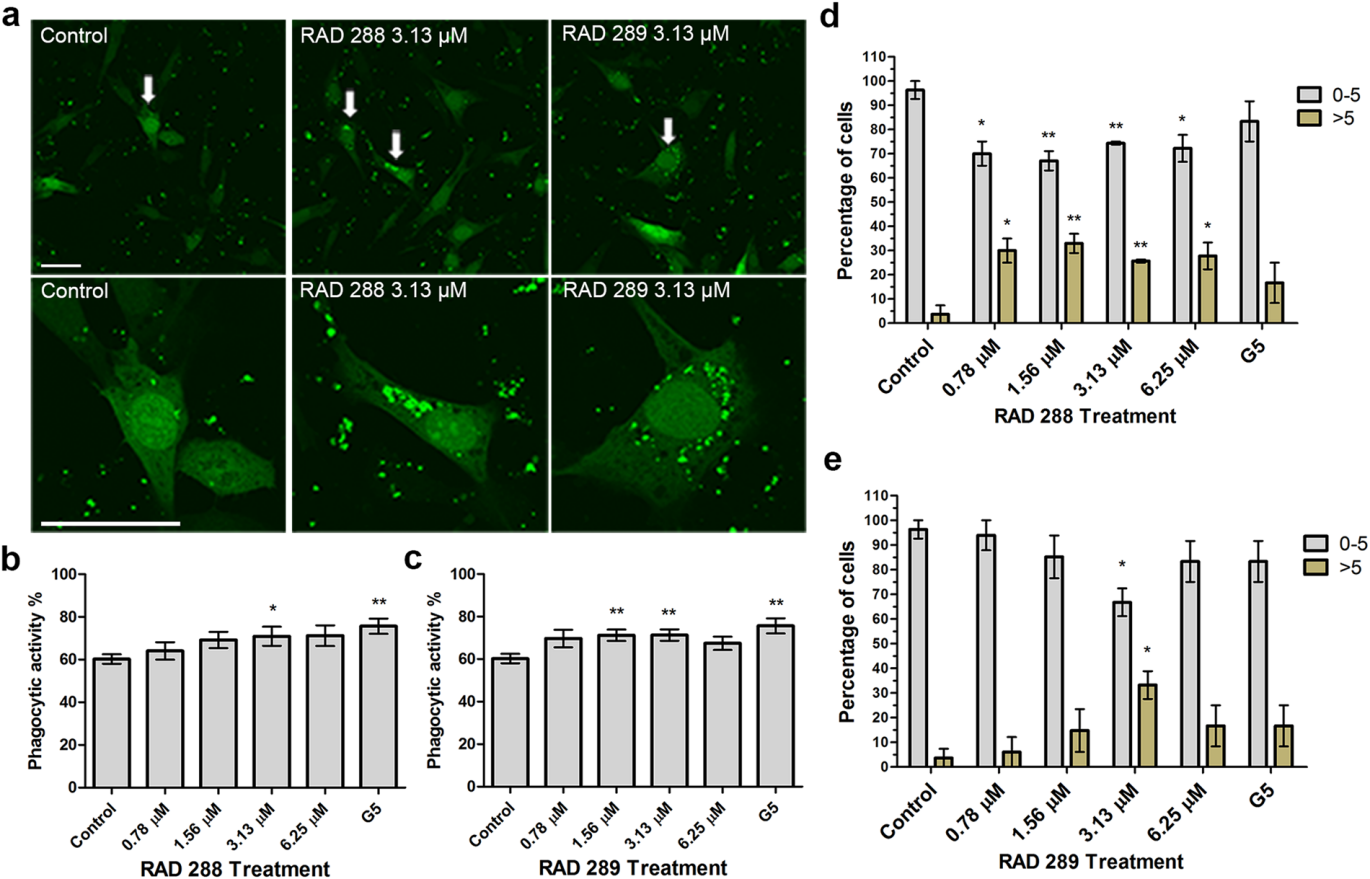
Effect of RAD288 and RAD289 on the phagocytic activity of mOECs. (**a**) Representative images showing RAD288 and RAD289 stimulating phagocytosis of neuron debris compared to control. Scale bar = 50 µm. (**b**) Phagocytic activity of mOECs after treatment with several concentrations of RAD288. (**c**) Phagocytic activity of mOECs after treatment with several concentrations of RAD289. (**d**) Proportion of mOECs phagocytosing 0 to 5 particles of neuronal debris and more than 5 particles of neuronal debris after treatment with several concentrations of RAD288. (**e**) Proportion of mOECs phagocytosing 0 to 5 particles of neuronal debris and more than 5 particles of neuronal debris after treatment with several concentrations of RAD289. Values are presented as means ± SEM of three independent experiments. **p < 0.01, *p < 0.05, Student’s t-test.

To investigate whether RAD288 and RAD289 stimulated the capacity of mOECs to engulf neuron debris, mOECs were incubated with axonal debris and the compounds for 24 h. The phagocytic activity of mOECs was defined as their capacity to engulf cell debris particles. The number of mOECs containing 0 to 5 debris particles or more than 5 debris particles was then determined. The number of particles of axonal debris taken up by mOECs treated with RAD288 and RAD289 was significantly increased as compared with control (Fig. 7a). After the number of cells with debris per treatment was determined, it was found that the phagocytic activity of mOECs increased significantly by 17% (p < 0.05) after treatment with RAD288 at 3.13 µM (Fig. 7b). In addition, an 18% (p < 0.01) increase (78% cells contain debris) in the phagocytic activity of mOECs after treatment with RAD289 at concentrations of 1.56 and 3.13 µM was observed in comparison with the control DMSO (Fig. 7c). No effect was noticed after treatment with other concentrations of RAD288 and RAD289 (p > 0.05). G5 supplement stimulated the phagocytosis of neuronal debris by 26% (p < 0.01) when compared with control (Fig. 7b,c). RAD288 and RAD289 also affected the proportion of debris engulfed by mOECs. We counted the number of particles of debris contained within each cell and allocated the cells to two groups: 0–5 particles, or more than 5 particles. After treatment with 0.78, 1.56, 3.13 and 6.25 µM of RAD288, the percentage of mOECs taking up 0 to 5 debris particles decreased by 26% (p < 0.05), 29% (p < 0.01), 25% (p < 0.01) and 24% (p < 0.05) respectively in comparison with the control. In contrast, a 26% (p < 0.05), 29% (p < 0.01), 25% (p < 0.01) and 24% (p < 0.05) increase in the proportion of mOECs engulfing more than 5 particles of neuronal debris after exposure to 0.78, 1.56, 3.13 and 6.25 µM of RAD288 respectively was also observed (Fig. 7d). Treatment with RAD289 at 3.13 µM was associated with a 30% (p < 0.05) decrease in the percentage of mOECs engulfing 0 to 5 debris particles, and a 30% (p < 0.05) increase in the proportion of mOECs taking up more than 5 neuronal debris particles as compared with the negative control (Fig. 7e). Therefore, the phagocytic activity of mOECs was significantly enhanced after treatment with both RAD288 and RAD289.

### RAD288 and RAD289 enhance the cell growth in 3D OEC sph*e*roids

**Figure 8:**
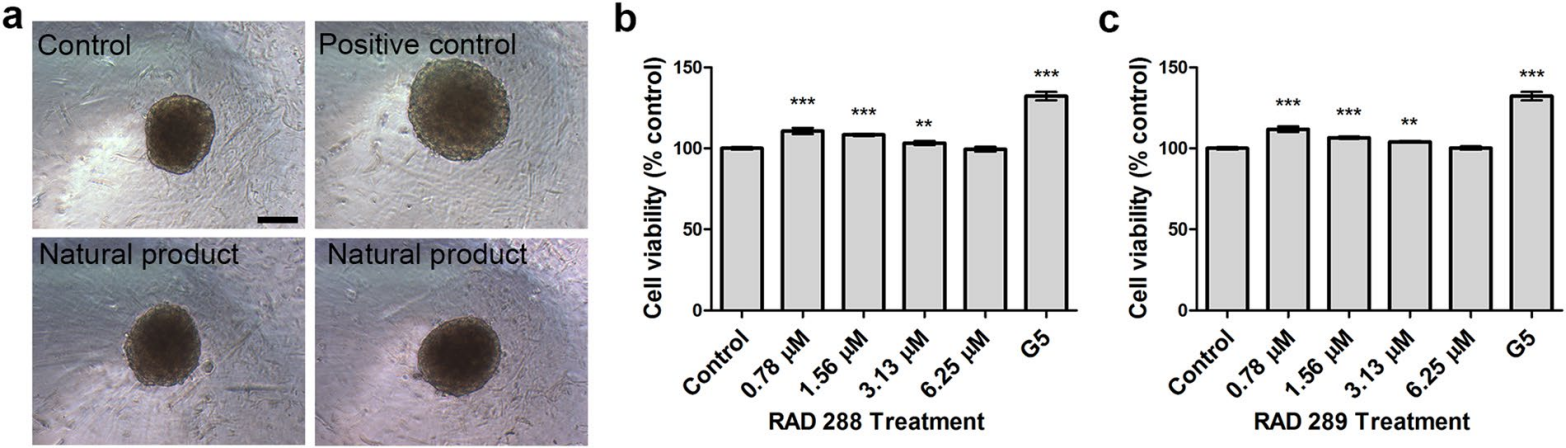
Effect of RAD288 and RAD289 on 3D mOECs spheroids. (**a**) Representative images of mOECs spheroids after drug exposure. Scale bar = 100 µm. (**b**) Cell viability of mOECs spheroids exposed to 0.2% DMSO (Control), 1% G5 supplement and RAD288 (0.78–6.25 µM) using the resazurin metabolic activity indicator. (**c**) Cell viability of mOECs spheroids exposed to 0.2% dimethyl sulfoxide (Control), 1% G5 Supplement and RAD289 (0.78–6.25 µM) using the resazurin metabolic activity indicator. Triplicate wells were used in three separate experiments, mean ± SEM, ***p < 0.001, **p < 0.01, Student’s t-test.

The use of 3D cell models is an emerging approach in tissue repair, as cells tend to have better survival rates and functions in 3D cell culture^19^. Consequently, the effect of RAD288 and RAD289 on the viability of 3D mOEC spheroids was investigated. An 11% (p < 0.001), 8% (p < 0.001) and 3% (p < 0.01) increase in cell viability was observed after treatment with RAD288 at 0.78, 1.56 and 3.13 µM respectively (Fig. 8a,b). Very similar results were shown after exposure to RAD289. Indeed, the viability of 3D mOECs increased by 11% (p < 0.001), 6% (p < 0.001) and 3% (p < 0.01) after exposure to RAD289 at 0.78, 1.56 and 3.13 µM respectively (Fig. 8a,c). Treatment with G5 supplement induced a 30% (p < 0.001) increase in 3D mOEC spheroid viability.

## Discussion

In this study we showed that the serrulatane diterpenoids 3-acetoxy-7,8-dihydroxyserrulat-14-en-19-oic acid (RAD288) and 3,7,8-trihydroxyserrulat-14-en-19-oic acid (RAD289) stimulated the proliferation and phagocytic activity of OECs, but that only RAD288 positively stimulated migration. The stimulatory effect of these natural compounds was cell-type specific, as there was no effect on the proliferation of Schwann cells, a glial cell type closely related to OECs.

While OECs are being trialled for cell transplantation to enhance neural repair, there are some limiting factors that could be addressed in order to improve therapeutic outcomes. Prior to transplantation, it is necessary to obtain sufficient numbers of cells during the *in vitro* expansion phase of OEC cultures. When using autologous cells, the slow proliferation of OECs means that the cells must be maintained *in vitro* for several weeks during which time their morphology and characteristics may alter^21^. Therefore strategies that increase the proliferation of OECs would result in more cell numbers in a shorter period and hence reduce the potential for undesirable changes in cell quality or characteristics that occur with extended cultures. Growth factors are commonly used in the culture media and injury site to support growth of OECs. However, it has been shown that the expression of low-affinity nerve growth factor receptor is reduced rapidly in a normal cell culture medium^22^. Therefore, the effects of growth factors are attenuated after several passages. The positive control G5 Supplement contains a mixture of growth factors and compounds and thus while it can stimulate cell growth and activity *in vitro*, the inclusion of growth factors may also lead to attenuation of effect over time. Moreover, the large molecular size and side effects associated with growth factors diminish their potential as good drug candidates for SCI treatments. The benefit of using a single compound such as RAD288 or RAD289 to stimulate OEC growth and activity *in vitro* is that it simplifies the production process to generate sufficient cells prior to transplantation. A simple production process, without the need of using complex mixtures of various factors such as G5 Supplement, would facilitate the approvals process needed for good manufacturing production of the cells for human transplantation.

Several natural products have been found to enhance cell proliferation in stem cell-based therapies. Indeed, *Radix aconiti lateralis preparata* has been found to increase 20% the proliferation rate of mouse bone marrow mesenchymal stem cells compared to untreated cells^23^. In addition, blueberry and green tea extracts promote an approximately 30% proliferation rate of human bone marrow cells^24^. Both RAD288 and RAD289 promoted OEC viability and proliferation by 25% and 40% respectively over 24 h. When used to accelerate the production of a certain number of cells for transplantation, RAD288 and RAD289 would decrease the time needed in culture. In addition, medicinal chemistry will be used to improve potency and specificity of RAD288 and RAD289.

We showed that RAD288 and RAD289 induced an increase in nucleus size. Several factors can be responsible for changes in nucleus morphology, including DNA content, cytoplasmic volume, nucleocytoplasmic transport and nuclear structural components^25^. RAD288 and RAD289 change OEC morphology by increasing the cytoplasmic volume. RAD289 at 6.25 µM has strong effects both on cell proliferation and cytoplasmic volume. Doubling of the cell mass is required in cell division^26^ and large cells generate more mRNA and are associated with higher transcriptional output^27^ enhancing biosynthetic capacity. Therefore, RAD288 and RAD289 may enhance the accumulation of proteins in OECs leading to a faster cell division rate.

RAD288 enhances the migration ability of OECs, which is a crucial factor in cell transplantation therapy. It has been found that OECs migrate and associate with extending axons into the lesion site of the spinal cord^28^. In addition, OECs were found to migrate along the damaged axon (1.5 cm/6 weeks) and promote long-distance axonal regeneration in adult rat spinal cords^29^. Moreover, OECs were also observed in the damaged site of the axon, and an OEC-rich environment increased axon extension in a mouse olfactory bulbectomy model^8^. Therefore, the “repair” function of OECs may require close contact with damaged neurons. However, in several cases, transplanted OECs show limited migration^30,31^. It has been shown that 14 days after injury, reactive astrocytes will seal the lesion site to form the glia scar, which may induce inhibition of axonal regeneration^32^. As RAD288 stimulates the migration of OECs, it could potentially enhance the migration OECs *in vivo* after transplantation into the injured spinal cord leading to a reduction in scar tissue formation, which would subsequently facilitate improved axon growth. In contrast to RAD288, the structurally similar RAD289 had no effect on the migration of OECs, which indicates that the binding and activation of the receptor that results in stimulating the migration of OECs is quite specific. The apparent cell migration response in a scratch assay can be due to both migration and proliferation, as the generation of new cells can drive cell movement into the cell-free area. However, RAD289 had no effect on migration while it did stimulate proliferation, thus if proliferation was a driver of apparent migration in the scratch assay there would have been an observed increase in migration, but no stimulation of migration was detected. Thus for RAD288, it is likely that any effect of proliferation is small and that the observed change in migration is therefore not just due to proliferation. From a chemical perspective, RAD288 contains an acetoxy moiety rather than the hydroxy group which is present in RAD289. This small chemical difference [i.e. one acetyl unit (CH_3_CO-)] appears to be responsible for the significant difference in OEC activity. While this acetyl group may have its effect via additional binding to a particular receptor in the biological system, the effect may also be due (in part or entirely) to the increase in lipophilicity of RAD288 compared to RAD289. Calculated LogP values using CambridgeSoft ChemDraw^**®**^ Ultra software for both molecules (CLogP = 4.05, RAD288; CLogP = 3.75 RAD289) show that there is a slight increase in the lipophilic physicochemical properties of RAD288, which may aid in cell penetration.

Phagocytosis is essential for clearing neuron debris to support axonal regrowth. Microglia plays an important role in removing tissue debris in central nervous system (CNS) injury and Alzheimer’s disease^33,34^. However, microglia may only have high efficiency at processing CNS debris in the early stage of SCI^35^ and slower clearance of CNS debris may affect axonal regeneration^36^. Therefore, removing CNS debris as rapidly as possible will facilitate neuron regrowth. OECs are highly phagocytic cells^37^ and the phagocytic activity of OECs has been previously shown to be stimulated by the natural product curcumin^38^. Similarly, we have now also observed that RAD288 and RAD289 stimulated the phagocytic activity and increased the number of neuronal debris engulfed by OECs. Therefore these natural compounds could be used to promote a suitable environment for axonal regeneration^38^.

3D models have several advantages for cell growth compared to 2D monolayer cell culture. For instance, cell-cell communication is maintained^39^ during transplantation process, so cell spheroids can be directly injected to the injured site or seeded on a biomaterial^40,41^. In addition, cellular functions such as cell receptor gene expression are different in 3D culture^42,43^, and cell growth in 3D better mimics the organization and metabolic characteristics of tissue. Moreover, 3D models promote cell viability leading to an increase in cell survival after implantation^44^. As approaches for preparing cells for transplantation now include embedding cells within hydrogels or other three-dimensional constructs, a compound incorporated into the gel/construct that diffuses out over time would therefore be useful for stimulating the OEC growth and activity after transplantation. We showed that G5 Supplement, RAD288 and RAD289 stimulated cell viability of 3D OEC spheroids. However, G5 is an expensive mixture of growth factors, hormones and transferrin, and is consequently not suitable for further drug development as the various components pose a range of potential side-effects. In comparison, RAD288 and RAD289 are pure small molecules that have the potential to enhance the transplantation process of 3D OEC spheroids. Medicinal chemistry on either RAD288 or RAD289 could be used in the future to potentially improve stability, selectivity, and *in vivo* efficacy or to reduce any potential side-effects associated with these compounds.

In conclusion, we showed that the serrulatane diterpenoids RAD288 and RAD289 enhanced proliferation and phagocytic ability of mOECs and that only RAD288 stimulated migration of the cells. In contrast, RAD288 and RAD289 had no effect on Schwann cell viability suggesting that they may have cell-type specific effects on OECs. Therefore, these natural products may have high potential for improving the use of OECs for transplantation therapy for neural repair.

## Methods

### Cell culture conditions

GFP-expressing immortalized mouse OECs (mOECs) were a gift from Professor Filip Lim (Universidad Autónoma de Madrid, Spain) obtained from primary cultures of olfactory bulb ensheathing glia from GFP-expressing mice (C57BL/6-Tg(ACTB-EGFP)1Osb/J, Jackson Laboratory, Bar Harbor, USA)^45,46^. Cells were maintained in complete media containing Dulbecco’s Modified Eagle Medium/Nutrient F-12 (DMEM/F12) supplemented with 10% fetal bovine serum (FBS, Bovogen) and 0.5% gentamicin. Dorsal root ganglion Schwann cells, isolated from S100ß-DsRed transgenic mice^47^, were cultured in complete media containing Dulbecco’s Modified Eagle Medium (DMEM) supplemented with 10% FBS and 0.5% gentamicin. mOECs and Schwann cells were incubated at 37 °C and 5% CO_2_ until 80–90% confluence was reached.

### Natural products

3-Acetoxy-7,8-dihydroxyserrulat-14-en-19-oic acid (RAD288) and 3,7,8-trihydroxyserrulat-14-en-19-oic acid (RAD289) were isolated from the air-dried aerial parts of *Eremophila microtheca* using a previously published separation protocol^17^. The compounds were determined to be at least 95% pure by ^1^H NMR and LC-MS. A 10 mM stock solution of RAD288 and RAD289 was prepared in DMSO for subsequent experiments. CambridgeSoft ChemDraw® Ultra software version 14.0.0.118 was used for LogP calculation (www.cambridgesoft.com).

### Positive control for assays

G5 Supplement (ThermoFisher Scientific) is designed for use with glial cell cultures and provides robust stimulation of glial cell growth and activity. It contains a mixture of biotin (100 mg/L), basic FGF (0.5 mg/L), EGF (1.0 mg/L), human transferrin (5000 mg/L), insulin (500 mg/L), hydrocortisone (0.36 mg/L) and selenite (0.52 mg/L).

### Cell proliferation assay

mOECs or Schwann cells were seeded at a density of 2000 cells per well in a 384-well microplate (Greiner). After 24 h, the culture media was removed and different treatments in complete media were added: (1) negative control: 0.2% DMSO, (2) positive control: 1% G5 supplement (100X), 0.2% DMSO, (3) concentrations from 0.02 to 12.5 µM of RAD288 and RAD289, 0.2% DMSO. To assess cell viability, after 24 h incubation with the different treatments, 5 µL of resazurin (500 µM, Sigma Aldrich) were added to each well leading to a resazurin final concentration of 50 µM. The plate was then incubated for 4 h at 37°C and 5% CO_2_. The fluorescent signal was quantified with an EnVision™ Multilabel (Perkin Elmer) plate reader at 535/595 nm. Then the cells were fixed in 4% PFA for 10 min. After fixation, cells were washed 3 times with PBS and stained with Hoechst (1:5000, Life Technologies, New Zealand) for 10 min and then washed 3 times with PBS. Cells were imaged automatically using Operetta^™^ (PerkinElmer), a high content imaging system using a 20X high numerical aperture objective lens. Individual cell segmentation and cell count were performed using the Harmony 3.5.2^®^ software.

### Cellular morphology analysis

mOECs were seeded at a density of 2000 cells per well in a 384-well microplate (Greiner). After 24 h, the culture media was removed and different treatments in complete media were added for 24 h: (1) negative control: 0.2% DMSO, (2) positive control: 1% G5 supplement (100X), 0.2% DMSO, (3) concentrations from 0.78 to 6.25 µM of RAD288 and RAD289, 0.2% DMSO. Cells were then fixed in 4% PFA. After fixation, cells were washed 3 times with PBS and stained with Hoechst (1:5000, Life Technologies, New Zealand) and CellMask Deep Red (1:5000, Life Technologies, USA) in PBS for 10 min before being washed 3 times with PBS. Cells were imaged by the Operetta^™^ (PerkinElmer) and 20 z-stack slices were acquired at 1 µm intervals. Cell volume was determined by analysing approximately 3,000–3,500 cells per well, using the Harmony 3.5.2® software. 3D reconstruction was generated by confocal microscopy (Olympus, FV1000) and Imaris 3.0.

### Cell migration assay

mOECs were seeded at a cell density of 15,000 cells per well into a 96-well microplate (Nunclon) in complete media. After the cells reach ~95% confluence, a p200 pipet tip was used to create a scratch of the cell monolayer. The width of scratch was approximately 700 µm. Then, the culture media was removed and different treatments in complete media were added for 24 h: (1) negative control: 0.2% DMSO, (2) positive control: 1% G5 supplement (100X), 0.2% DMSO, (3) concentrations from 0.78 to 6.25 µM of RAD288 and RAD289, 0.2% DMSO. HEPES (4-(2-hydroxyethyl)-1-piperazineethanesulfonic acid, 1%) was added to culture medium to maintain a physiological pH range (7.2–7.4) in room air (0.03% CO_2_ environment). The migration behaviours of mOECs after treatments were recorded every 10 min for 24 h using time-lapse microscopy (Olympus IX81). Images were analysed using Image J to assess cell migration.

### Phagocytosis assay

mOECs were seeded at a cell density of 2000 cells per well in a CellCarrier-384 Ultra microplate (PerkinElmer) in 50 µL complete media. After 24 h, the culture media was removed and different treatments were added for 24 h: (1) negative control: 0.2% DMSO, (2) positive control: 1% G5 supplement (100X), 0.2% DMSO, (3) concentrations from 0.78 to 6.25 µM of RAD288 and RAD289, 0.2% DMSO. Neuronal debris were obtained from OMP-ZsGreen fluorescent protein reporter transgenic mice^48^ as previously described^38^ and added at a concentration of 50 µg/mL. After 24 h incubation, the cells were fixed using 4% PFA. Cells were imaged by confocal microscopy (Olympus, FV1000) and the number of particles of debris inside the cells was then counted. The number of cells containing no debris, less than 5 debris particles and more than 5 debris particles was also determined by confocal microscopy (Olympus, FV1000), n = 100.

### Multicellular 3D spheroids

mOECs were seeded in Corning 384-well spheroid microplates at a cell density of 5000 cells per well in 50 µL complete media. After 24 h, 25 µL of culture medium was removed and different treatments in complete media were added: (1) negative control: 0.2% DMSO, (2) positive control: 2% G5 supplement (100X), 0.2% DMSO, (3) concentrations from 0.78 to 6.25 µM, 0.2% DMSO of RAD288 and RAD289. After 24 h incubation with the different treatments, 5.5 µL of resazurin (500 µM, Sigma Aldrich) was added to each well leading to a resazurin final concentration of 50 µM. The plate was then incubated for 4 h at 37 °C and 5% CO_2_. Cells were imaged using the Olympus CKX41.

### Statistical analysis

Kolmogorov–Smirnov test was used to determine if the data appeared to follow a normal distribution. Statistical significances were assessed using Student’s t-test or Mann-Whitney U test. Statistics and graphical analysis were performed using Graphpad Prism 6 ™ software.

### Data availability

We have provided in the manuscript all the necessary data to support our results. If referees consider any more data is necessary we will be happy to provide it in the revised manuscript.
